# The impact of schizotypy on quality of life among adults with autism spectrum disorder

**DOI:** 10.1186/s12888-022-03841-2

**Published:** 2022-03-19

**Authors:** Albin Klang, Britta Westerberg, Mats B. Humble, Susanne Bejerot

**Affiliations:** 1 School of Medical Sciences, Örebro University, Örebro, Sweden; 2 University Health Care Research Centre, Region Örebro County, Faculty of Medicine and Health, Örebro University, P.O. Box 1613, University Hospital, S-701, Örebro, Sweden

**Keywords:** Schizotypy, Autism spectrum disorder, Schizotypal personality disorder, Quality of life

## Abstract

**Background:**

Autism spectrum disorder (ASD) and schizotypal personality disorder can be difficult to distinguish. Deficits in social relationships and social interaction, present in both conditions, are known to impair quality of life. The aim of the present study was to investigate if schizotypal symptoms affect quality of life among adults diagnosed with autism spectrum disorder and to study the association between schizotypy and autistic traits among them.

**Methods:**

Participants diagnosed with autism spectrum disorder (*n* = 110) completed questionnaires exploring schizotypy (Schizotypal Personality Questionnaire – Brief Revised (SPQ-BR)), autistic traits (The Ritvo Autism, Asperger Diagnostic Scale-Revised Screen 14 items), anxiety and depression (The Hospital Anxiety and Depression scale) and quality of life (Brunnsviken Brief Quality of Life Scale and the European quality of life index version 5D).

**Results:**

Schizotypy was found to be associated with anxiety, depressive and autistic symptoms, and poor quality of life. Although schizotypy was a predictor for impaired quality of life, this relationship was mediated by symptoms of anxiety and depression, plausibly inherent to autism. Autistic traits were positively associated with all higher order constructs of the SPQ-BR, i.e. positive and negative schizotypy, disorganization and social anxiety, as well as with poor quality of life.

**Conclusions:**

There is considerable overlap between schizotypy and autism that needs to be considered in research. Prominent schizotypal traits in people with ASD may constitute an endophenotype coinciding with a particularly poor quality of life.

**Trial registration:**

ClinicalTrials.gov identifier: NCT03570372: Internet-based Treatment for Adults with Autism Spectrum Disorder (MILAS).

## Background

In Autism Spectrum Disorder (ASD), psychiatric comorbidity is often present [26, 30]. The most common comorbidities among people with ASD who have average intelligence are anxiety disorders, depression and attention deficit hyperactivity disorder [33]. Psychiatric disorders are well known predictors for poor quality of life, and depression and anxiety are important factors [55]. Accordingly, concurrent psychiatric diagnoses predict reduced quality of life in ASD [37], however quality of life is generally impaired among people with ASD, regardless of psychiatric comorbidities [53]. Examples of areas associated with quality of life are life satisfaction, perceptions of social relationships, physical health, economic status and overall functioning in daily activities and work [27], areas regularly affected in ASD.

According to the diagnostic hierarchies of DSM-5 and ICD-11, personality disorder diagnoses cannot be established in individuals with ASD, however symptoms of personality disorder can nevertheless concur. Moreover, it can be challenging to distinguish ASD from personality disorders because of a considerable phenotypical overlap [34]. Especially schizotypal personality disorder and ASD share a number of traits [13, 15, 47].

Notably, traits labelled schizotypy can be found to different degrees, not only in patients but also within the general population. Although schizotypy is far from being a uniform construct [29], it is typically characterised by eccentric behaviours, odd speech, social anxiety and paranoid ideas [43, 56]. Psychotic symptoms such as ideas of reference and hallucinations may also occur, but to a lesser extent than in schizophrenia [2, 57, 58].

Individuals diagnosed with ASD may exhibit schizotypal traits, and individuals diagnosed with any schizophrenia spectrum disorder may show traits suggestive of ASD [3, 7, 13, 34, 47]. In both conditions deficits in how to build relationships and handle social interactions are prevalent, as well as social anxiety, odd behaviours and perceptual aberration [40]. However, according to the DSM-5, ASD is ranked hierarchically higher than personality disorders, i.e., if the conditions coexist, only the ASD diagnosis should be set, while according to ICD-11, schizotypal disorder is ranked above ASD. Both conditions can be viewed as dimensional disorders and intertwined [8, 19, 36, 59] with sub-clinical symptoms extending into the general population [16, 46]. Both conditions affect several areas of functioning and influence quality of life negatively, also among non-clinical adults [9, 42]. Accordingly, not only autistic traits but also pronounced schizotypal traits may indicate poor life satisfaction. However, to our knowledge no study has examined to what extent schizotypal traits affect quality of life in individuals with ASD.

The primary aim of this study was to investigate quality of life in individuals with ASD and to what extent it is affected by schizotypal, autistic, anxious and depressive symptoms. We hypothesized that high scores on assessments of schizotypy and autism negatively affect the perceived quality of life but may be mediated by anxiety and/or depression. A secondary aim was to investigate the strength of the correlation between ASD symptoms and schizotypy in individuals with ASD.

## Methods

### Participants

A total of 114 individuals across Sweden were enrolled to take part in a brief internet-based cognitive behavioural therapy research study, MILAS, aiming to evaluate whether the treatment improves quality of life in individuals with ASD. Participants were recruited through advertisements on supportive facilities for autism, webpages directed towards autism, other health care facilities and social media. Each participant had to state having been diagnosed with ASD by a medical doctor or a licenced psychologist and provide information on when and where this was done. The ASD diagnoses were confirmed in 70 out of the first 84 included participants by retrieving their individual medical records, which all endorsed their ASD diagnoses. Practical matters hindered confirmation in the remaining group, but we have no reason to expect any lesser reliability in these cases.

Inclusion and exclusion criteria were integrated with the questionnaires in a web survey, launched on 1177.se, a Swedish public platform for online healthcare. Inclusion criteria were age between 16 and 55 years and a diagnosis of ASD. Exclusion criteria were ongoing cognitive behavioural therapy, psychosis, intellectual disability, risk of suicide and change in medication within 6 weeks prior to inclusion. For the purpose of the current study, not completing the Schizotypal Personality Questionnaire was an additional exclusion criterion. Written informed consent was obtained from all participants. Procedures were approved by the Swedish Ethics Review Board.

### Measures

Prior to the therapeutic intervention, participants filled out a web-based survey including questionnaires pertaining to schizotypal traits, anxiety and depressive symptoms, autistic symptoms, and quality of life. Participants were also asked to respond to questions regarding gender, age, occupation, and any psychiatric diagnoses apart from ASD. The following questionnaires were included in this study:

#### The schizotypal personality questionnaire – brief revised (SPQ-BR)

Participants completed the SPQ-BR, a self-report scale measuring schizotypy [10]. This revised version retains 32 of the 74 items of the original SPQ [43], each item graded on a 5-point Likert-type scale. High scores indicate more schizotypal traits. The items are based on the DSM-III-R diagnostic criteria for schizotypal personality disorder. Subscales are applied as suggested by Davidson et al. [12]. Schizotypy, according to SPQ-BR, is conceptualized as a set of nine single order constructs that can be summarized by four more general higher order constructs: Positive schizotypy (i.e. Cognitive-perceptual, including the single order subscales Suspiciousness, Ideas of reference, Magical thinking and Unusual perceptions), Disorganized (including the single order subscales Eccentric behavior and Odd speech), Negative schizotypy (i.e. Interpersonal, including the single order subscales No close friends and Constricted affect) and Social Anxiety. The latter is regarded as simultaneously a single order subscale and higher order construct [12]. Although the use of summed scores is not supported by tests of uni-dimensionality and parallel items, according to the Davidson study, the sum is frequently reported in previous SPQ-BR research. The SPQ-BR has shown high to excellent internal consistency with a mean Cronbach’s alpha coefficient of 0.89 for the schizotypal trait subscale scores [6].

Because schizotypal traits vary greatly across countries, and no data on the Swedish version of SPQ-BR has yet been published, a convenience sample consisting of 41 health care workers (85% women, mean age 42 years) completed the questionnaire for the purpose of this study. Their SPQ-BR mean score was 41.9 (SD ± 10.57), considerably lower than the mean score of almost 70 in a non-clinical Spanish sample and over 80 in a US sample [16]. In our present study, the overall Cronbach’s alpha coefficient was 0.89.

#### The Hospital anxiety and depression scale (HADS)

HADS is a 14-item self-report screening tool to detect symptoms of depression and anxiety during the past week [60]. HADS includes two subscales, measuring depressive (HADS-dep), and anxiety symptoms (HADS-anx), respectively. Each item is scored between 0 and 3 with a maximum score of 21 for each subscale. The clinical cut off score is above 10, whereas a score of 6 or below excludes depression or anxiety disorder. Mean score in a non-clinical Swedish population was reported to be 3.98 on the depression subscale and 4.55 on the anxiety subscale [32]. In our study, the overall Cronbach’s alpha coefficient was 0.9 and for the anxiety and depression subscales, it was 0.84 and 0.83, respectively.

#### Ritvo autism and asperger diagnostic scale-revised 14 items screen (RAADS-14 screen)

The RAADS-14 Screen is an abbreviated version of 80-item RAADS-R, a self-rated instrument intended to measure traits typical for ASD in adults [45]. RAADS-14 Screen consists of 14 items divided into three subscales: Mentalizing deficits (7 items), Social anxiety (4 items) and Sensory reactivity (3 items). Each item is graded between 0 and 3 with a maximum score of 42. Higher scores indicate more symptoms related to autism. Mean score in a non-clinical Swedish population was 3, in a psychiatric non-autistic population 15, whereas an ASD population had a mean score of 32 [14]. The overall Cronbach’s alpha coefficient was 0.77 in the present study.

#### The Brunnsviken brief quality of life scale (BBQ)

The BBQ is a 12 item self-report instrument developed to measure quality of life within six life areas (Leisure time, View on life, Creativity, Learning, Friends and Friendship, and View of self). Two items cover each area; one measures satisfaction while the other concerns the importance of that particular area. Each item is rated on a 5-point Likert-type scale with scores between 0 and 4. High scores indicate a good quality of life. The total score is obtained by multiplying the satisfaction and importance item for each area and then adding them. The maximum score is 92. The BBQ has been validated in healthy populations as well as in a large sample of individuals with social anxiety. Mean scores among a Swedish student population was 60.1 [31]. In the present study, the overall Cronbach’s alpha coefficient was 0.82.

#### European quality of life index version 5D, five level version (EQ-5D-5L)

EQ-5D-5L is an instrument used to describe health related quality of life. It consists of two parts, one descriptive (EQ-5D index) that comprises five dimensions: Mobility, Self-care, Usual activities, Pain/discomfort and Anxiety/depression, and one visual analogue scale (EQ VAS). The visual analogue scale is used as a complementary measure of health outcome [21]. The descriptive part includes 5 items, each representing one dimension of health and graded on a 5-point Likert scale. The dimensions generate a 5-digit code to create an index of health (ranging between 0 and 1), 0 equals death and 1 equal perfect health. The visual analogue scale EQ VAS is rated on a vertical axis, between 0 and 100, ranging from “the worst health you can imagine” to “the best health you can imagine”. Mean scores in a Swedish non-clinical adult population were 0.85 on the index scale and 83.3 on the visual analogue scale [25]. The overall Cronbach’s alpha coefficient for the EQ-5D-index in our study was 0.71.

### Data analysis

All personal data was anonymized. The participants were divided into two groups based on their mean SPQ-BR scores. The response rate was set at a minimum of 80% of the questions for inclusion in the study. For the remaining data, isolated missing items were imputed with the individual’s mean on related items. All scores derived from the symptom questionnaires were treated as continuous variables.

Chi-square test of independence was conducted to investigate if group affiliation was associated with gender, having a psychiatric comorbidity (yes/no) or having an occupation (occupation/studying vs neither). Spearman’s rank order correlation was performed to assess whether there was an association between the outcomes quality of life (BBQ) and health related quality of life (EQ-5D-5 L) in relation to SPQ-BR scores, for associations between SPQ-BR and RAADS-14, and for SPQ-BR subclass analyses. Spearman’s rank order was used since most of the data was not normally distributed.

Hierarchical regression analyses to estimate the relationships between variables were conducted in a two-step manner. For each step, three models were constructed, with BBQ, EQ-5D-index or EQ VAS as dependent variables, respectively. In the first step, independent variables were age, gender, and SPQ-BR. In the second step, the sum of the two HADS subscales (HADS-tot) was included as an independent variable to test the influence of depression and anxiety, since these are known contributors to impaired quality of life. To investigate whether or not the HADS-tot was a mediator between SPQ-BR and the dependent variables, bootstrapping analyses were conducted (SPSS macro Hayes Process v.3.5) [20]. This macro provides a total effect score, i.e., the effect of SPQ-BR on the dependent variables without considering the presence of a potential mediator, and a mediation model, where the effect mediated by HADS-tot is separated from the direct effect of SPQ-BR on the dependent variables (i.e., the quality-of-life measures). The mediation model illustrates the direct effect of SPQ-BR on the dependent variable when the mediator is disengaged, and the indirect effect of SPQ-BR, i.e., how much SPQ-BR affects the dependent variable through mediation. Correction for multiple testing was not made. SPSS version 27 was used for all analyses.

## Results

### Patient characteristics

**Fig. 1:**
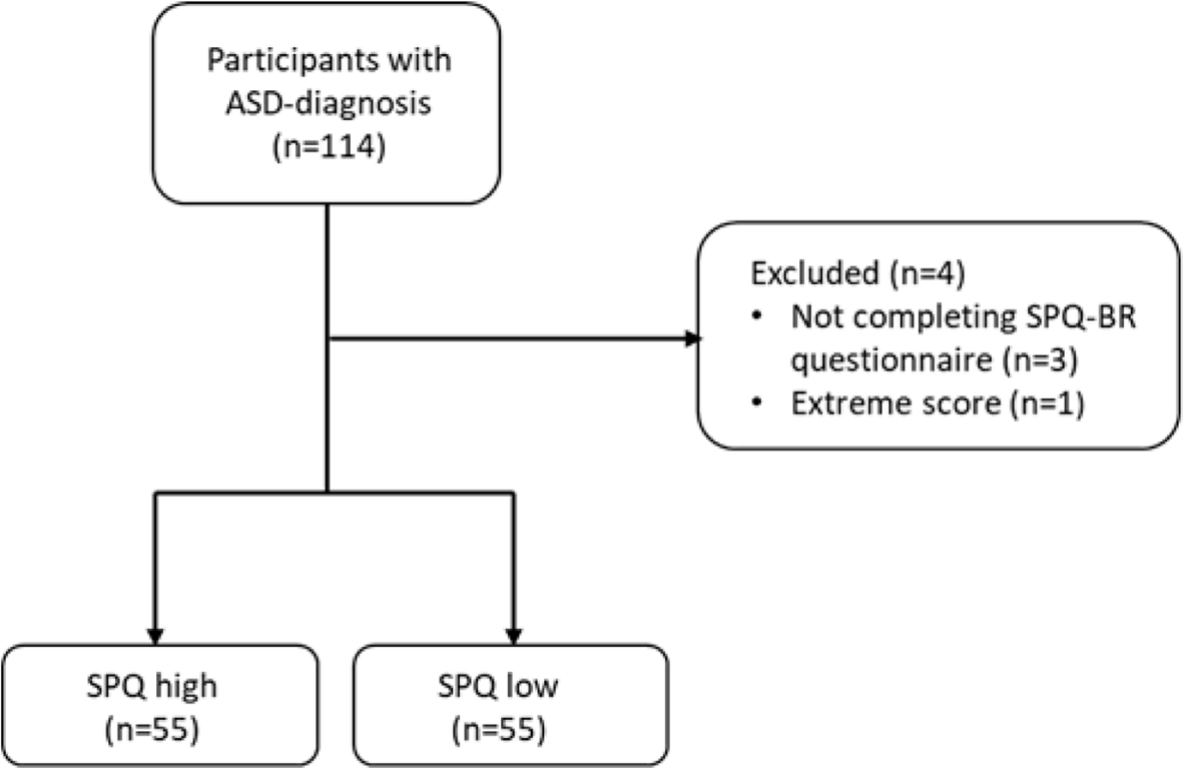
Flowchart of participants and the group distribution

After exclusions, 110 participants (70 women and 35 men; 5 reported themselves to be non-binary, transgender, or queer) were included in the study. Four individuals had been excluded, 3 due to not responding to the SPQ-BR questionnaire and another one due to extreme scores, see flowchart Fig. 1. In 8 cases missing SPQ-BR data was imputed (one missing item in seven cases and 3 in one case). The mean SPQ-BR score was not affected by gender (SPQ-BR scores: 67.2/64.8/71.4 for women/men/other respectively). All participants were between 17 and 56 years with a mean age of 32.6 ± 9.6 years, and only one was younger than 18 years. Among 109 of the participants, 50.9% stated to have a daily activity either as employed (*n* = 31), student (*n* = 12) or participating in community based daily activity (*n* = 13), while the remaining were job seekers (*n* = 14), on sick leave (*n* = 27) or unemployed (*n* = 12). 60 participants reported at least one psychiatric comorbidity, 11 did not know if they had a psychiatric comorbidity, and 39 stated ASD to be their only diagnosis.

#### The SPQ high and SPQ low group

By dividing the participants according to their median SPQ-BR score, a SPQ low group and a SPQ high group were constructed, each including 55 individuals**.**

The SPQ high group reported lower scores on the BBQ, EQ-5D-index, and EQ VAS than the SPQ low group. Furthermore, analyses showed higher mean scores on the RAADS-14 Screen and the HADS subscales in the SPQ high group compared to the SPQ low group; see Table 1**.**

**Table 1:** Mean values of demography and questionnaires reported by the high versus low schizotypy groups

				*t*-test		Mann Whitney U -test		
	ALL *n* = 110(SD)	SPQ high *n* = 55 (SD)	SPQ low n = 55 (SD)	*t*	*df*	U	Z	*p*
Gender, M/W/O	35/70/5	17/35/3	18/35/2	/	/	/	/	.89
Age, mean yrs	32.6 (9.6)	34.0 (10.3)	31.2 (8.7)	/	/	1303	−1.3	.21
Comorbidity, % yes	53.5	62	47	/	/	/	/	.08
Employed or student, % yes	51.4	47.3	56.0	/	/	/	/	.39
RAADS-14	29.3 (9.5)	34.0 (5.2)	24.7 (8.3)	−7.0	90.6	/	/	<.001
EQ VAS	56.7 (22.5)	49.0 (23.6)	64.4 (19.2)	/	/	922.5	−3.5	<.001
EQ-5D index	0.6 (0.3)	0.5 (0.3)	0.7 (.2)	/	/	997.0	−3.1	.002
BBQ	40.0 (21.8)	33.0 (19.4)	47.1 (21.2)	3.7	107.2	/	/	<.001
HADS-dep	7.4 (4.2)	9.1 (3.8)	5.7 (3.9)	/	/	794.0	−4.3	<.001
HADS-anx	11.6 (4.8)	14.0 (3.7)	9.3 (4.7)	/	/	629.0	−5.3	<.001
SPQ-BR	66.6 (18.3)	80.8 (11.7)	52.4(11.2)	/	/	<.001	−9.0	<.001

*M *men, *W *women, *O *other gender, *BBQ *Brunnsviken Brief quality of life scale, *EQ VAS *dimensional part of EQ-5D-5L, *EQ-5D index *descriptive part of EQ-5D-5L, *HADS-dep* depression subscale of The Hospital Anxiety and Depression scale, *HADS-anx *anxiety subscale of The Hospital Anxiety and Depression scale, *RAADS-14 *The Ritvo Autism and Asperger Diagnostic Scale-Revised 14 items, *SPQ-BR *The Schizotypal Personality Questionnaire – Brief Revised

For gender, psychiatric comorbidity and occupation, the *p* values are derived from χ^2^ analyses. Significance was set at *p* < 0.05

#### Bivariate correlations between measures of schizotypy, quality of life and autism

The correlations between SPQ-BR with subscales and measures of quality of life are given in Table 2. There were moderate negative correlations between SPQ-BR-total and BBQ, EQ-5D-index and EQ VAS (*rho* = −.44, −.39 and − .41, respectively, all *p* < .001). Slightly lower associations were found between RAADS-14 Screen and these same measures (*rho* = −.40 (*p* < .001), −.25 (*p* = .009) and − .24 (*p* = .01), respectively). There was a positive correlation between SPQ-BR and RAADS-14 Screen (*rho* = .58, *p* < .001).

**Table 2:** Correlations between the SPQ-BR-32 with its subscales and the RAADS-14 Screen with subscales and the measures of quality of life

		Ritvo Autism and Asperger Diagnostic Scale-14 Screen, RAADS-14 Screen				Quality of Life Measures		
		Mentalization deficit	Social anxiety	Sensory reactivity	*RAADS-14 Total*	*BBQ*	*EQ-5D index*	*EQ5 VAS*
Schizotypal Personality Questionnaire, SPQ-BR-32	**Cognitive-Perceptual**	.26	.29	***.33***	***.36***	−.27	***−.31***	−.26
	*Ideas of Reference*	.16	.21	.27	.25	−.21	−.18	−.15
	*Suspiciousness*	***.39***	***.39***	.22	***.45***	***−.41***	−.24	−.27
	*Magical Thinking*	- .02	- .06	.21	.02	.04	−.23	−.12
	*Unusual Perception*	.26	.18	.19	.29	−.23	−.20	−.16
	**Interpersonal**	***.45***	***.50***	.07	***.48***	***−.51***	−.24	−.26
	*No Close Friends*	***.39***	***.45***	.13	***.44***	***−.49***	−.25	−.22
	*Constricted Affect*	***.40***	***.42***	.02	***.40***	***−.38***	−.17	−.21
	**Social Anxiety**	***.33***	***.50***	***.51***	***.53***	***−.32***	***−.33***	−.28
	**Disorganized**	***.33***	.23	***.32***	***.39***	−.23	−.22	***−.31***
	*Eccentric Behaviour*	.30	.29	.27	***.37***	−.15	−.16	−.15
	*Odd Speech*	.27	.09	.25	.28	−.24	−.20	***−.33***
	***SPQ-BR-32 Total***	***.46***	***.49***	***.41***	***.58***	***−.44***	***−.39***	***−.41***

Spearman rank-order correlations: *Rho* coefficients are given, *N* = 110, *p* ≤ .001 for all bold, italic numbers

*BBQ *Brunnsviken Brief Quality of Life Scale, *EQ-5D *EQ-5D-5L, *VAS *visual analogue scale

Among the subscales of SPQ-BR, those included in Positive schizotypy (Cognitive-Perceptual) were only weakly correlated with those included in Negative schizotypy (Interpersonal), except for Suspiciousness which correlated with No close friends (*rho* = .39). On the other hand, the higher-order constructs Positive schizotypy and Disorganization were highly correlated with each other (*rho* = .59), mainly driven by the single order subscales Suspiciousness and Eccentric behaviour (*rho* = .57). Social anxiety correlated only with the single-order subscale Ideas of reference (*rho* = .37), and Magical thinking only with Unusual perception (*rho* = .41) (data not shown).

When all SPQ-BR subscales were correlated with the RAADS-14 subscales, Negative schizotypy (i.e. No close friends and Constricted affect) and SPQ Social Anxiety showed the highest correlations with RAADS-14 and its subscales (see Table 2). Among the remaining SPQ-BR subscales, only Suspiciousness, included in Positive schizotypy construct, showed a relationship with the RAADS- 14 subscales Mentalizing deficit and Social anxiety, while RAADS-14 Sensory reactivity only showed a relationship with SPQ-BR Social Anxiety. However, all higher-order SPQ constructs correlated with RAADS-14. For all here given correlations, *p* < .001.

#### Schizotypy as a predictor for quality of life

In the first step of the regression analyses, the models explained 18–26% of the variances of the dependent variables. SPQ-BR was a predictor for quality of life in all models while age and gender were predictors in some of them. In the second step of the multiple linear regression, three new models were constructed with HADS-tot added as an independent variable. The models then explained approximately 40–51% of the variance in the dependent variables. In the second model, SPQ-BR was a non-significant contributor, whereas HADS-tot was a significant predictor across all analyses. Gender and age remained predictive in some of the analyses. See Table 3a for the first step and Table 3b for the second step regression analyses without and with HADS as independent variable (Table 3a and b).

**Table 3:** First and second step of regression analyses without and with HADS as independent variable

Dependent variable	BBQ	EQ-5D index	EQ VAS
**a. First step, multiple linear regressions, n = 110**			
Independent variable:	Coefficient (unstandardized/standardized (*p*))		
Age	−.389/−.174 (.049)	−.001/−.041 (.650)	−.497/−.206 (.027)
Gender	−4.215/−.095 (.275)	−.148/−.283 (.002)	−4.210/−.087 (.339)
SPQ-BR	−.496/−.430 (< 0.001)	−.004/−.326 (<.001)	−.385/−.308(.001)
Constant	89.485(<.001)	1.036 (<.001)	101.737 (<.001)
R^2^	.259	.206	.175
F-ratio (*p*-value)	11.777(<.001)	8.739 (<.001)	7.153 (<.001)
**b. Second step, multiple linear regressions, n = 110**			
Independent variable:	Coefficients (unstandardized/standardized (*p*))		
Age	−.310/−.139 (.056)	<.001/−.009 (.905)	−.416/−.173 (.032)
Gender	−2.251/−.050 (.476))	−.127/−.243 (.002)	− 2.173/−.045 (.562
HADS-tot	−1.562/−.603 (<.001)	−.017/−.542 (<.001)	−1.621/−.579 (<.001)
SPQ-BR	−.117/−.101 (.239)	<.001/−.030 (.746)	.008/.007(.943)
Constant	89.889 (<.001)	1.041 (<.001)	102.156 (<.001)
R^2^	.505	.405	.402
F-ratio (*p*-value)	25.536 (<.001)	17.046 (<.001)	16.807 (<.001)

*HADS-tot *The combined Hospital Anxiety and Depression subscales, *SPQ-BR *The Schizotypal Personality Questionnaire – Brief Revised, *BBQ *Brunnsviken Brief quality of life scale, *EQ VAS *visual analogue scale of EQ-5D-5L, *EQ-5D index *descriptive part of EQ-5D-5L

Significance was set at *p* < 0.05

#### Depression and anxiety mediating quality of life

**Fig. 2:**
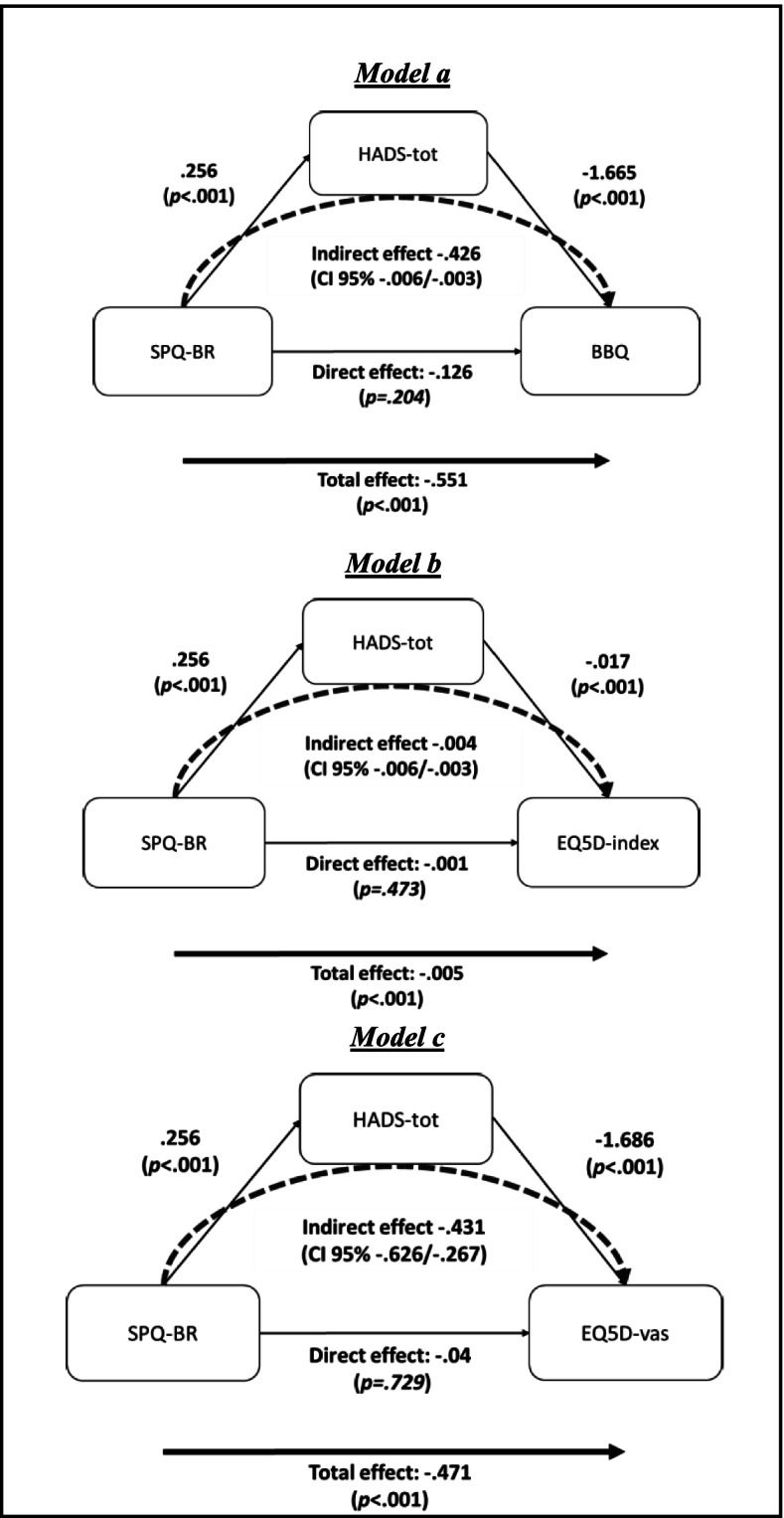
**a-c** Mediation models on the impact of schizotypy on the quality-of-life measures

Hayes process with 5000 bootstrapping samples was conducted with the quality-of-life measures BBQ, EQ-5D-index and EQ VAS as dependent variables, which resulted in three different models. In each model, we added HADS-tot as a potential mediator. In all models, SPQ-BR had a total effect on the dependent variable while the direct effect of SPQ-BR was nonsignificant. However, the indirect effect was statistically significant in all cases. Given that the direct effect in all the models was non-significant while the indirect effect was significant, the effect of SPQ-BR on the dependent variables BBQ, EQ-5D-index and EQ VAS, respectively, appeared fully mediated by HADS-tot. Figure 2a-c illustrates the total, indirect and direct relationships (with standardized regression coefficients) between SPQ-BR and the mediator (HADS-tot) on the dependent variables measuring quality of life.

### Post-hoc analysis of core schizotypy traits

In order to investigate core symptoms of schizotypy, independent of autistic traits, we performed a post hoc analysis. After excluding four of the SPQ-BR subscales (i.e., Suspiciousness, No close friends, Constricted affect, Social Anxiety) that were strongly associated with any of the RAADS-14 subscales (*p* < .001), the correlation between the total score of the five remaining SPQ-BR subscales (i.e. Ideas of reference, Magical thinking, Unusual perception, Eccentric behaviour, Odd speech) and RAADS-14 total score was diminished from rho = .58 to rho = .36 (*p* < .001). Moreover, the correlation with the RAADS-14 subscales Mentalizing deficits decreased from rho = .46 to rho = .27 (*p* < .005), the Social anxiety subscale from rho = .49 to rho = .23 (*p <* .02), and the Sensory reactivity subscale from rho = .41 to rho = .38 (*p* < .001). The relationship between the five SPQ-BR subscales and HADS, EQ-5D-index, EQ VAS and BBQ diminished similarly but remained significant (HADS, rho = .57 to rho = .43, *p* < .001; EQ-5D-index, rho = .39 to rho = .30, *p* < .005; EQ VAS, rho = .41 to rho = .31, *p* < .001; BBQ, rho = .44 to rho = .24, *p* < .02). The results indicate that Positive/cognitive-perceptual and Disorganised schizotypy, which lack obvious overlaps with autistic traits, still have an independent and negative effect on quality of life and life satisfaction in adults with ASD, however to a lesser extent than when schizotypy (defined by the SPQ-BR) also includes negative/interpersonal schizotypy.

## Discussion

In the present study, we have shown that a high level of schizotypy predicts a poor quality of life in adults with ASD. Moreover, we found that the negative effect of schizotypy was indirect and, apparently, largely mediated by anxiety and depressive symptoms. Although the causal direction of these relationships cannot be settled from our cross-sectional design, it seems highly plausible that more schizotypal traits result in poor quality of life, rather than the reverse. Our study is also one of the first to assess schizotypy in subjects with ASD. To our knowledge, only Spek & Wouters [50] and Barneveld et al. [3] have published on this, using SPQ in small samples consisting of 21 and 27 individuals with autism, respectively.

It is well known that adults with ASD experience low overall quality of life [22, 37, 53]. Compared to studies using the same quality of life measures as we did, our participants scored considerably lower than healthy controls [25, 31]. In addition, our participants reported lower quality of life when compared to autistic children [52]. Presumably, with age people gain insight on what to expect of life and subsequently become disillusioned, which is reflected by the low scores on quality-of-life measures. Expectedly, another contributor to poor quality of life was, apart from severity of autistic and schizotypy symptoms, psychiatric comorbidity. Mental health comorbidity predicts poor quality of life in people with ASD [37] and psychiatric comorbidity could even be viewed as a sign of overall severity [28], likewise linked to quality of life.

Many studies report strong correlations between anxiety and depression, and poor quality of life [18, 44, 48]. Depression is hypothetically a common pathway for quality of life, i.e., a mechanism by which all factors found to affect quality of life are mediated. In fact, depression has been shown to impact quality of life across conditions and influence life quality to a greater extent than physical illness does [24, 35, 49]. Our findings could be understood analogously. We found that a large part of the negative effect on quality of life, driven by schizotypal traits, was indeed mediated through depression and anxiety symptoms. Our participants presented high scores on the anxiety and depressive measure HADS, which was strongly associated with increase in schizotypy and autism scores. HADS is intended to assess state [32], but in ASD, HADS is perhaps rather a trait measure.

With an international outlook, the SPQ-BR scores reported by our participants may seem modest. However, to decide whether a particular score is high or not we should only compare with people of similar cultural background. One reason for the low scores among Swedes could be religion. Belief in God is positively correlated with positive schizotypal traits [11], and Sweden is considered as the least religious country in the world (https://worldpopulationreview.com). Furthermore, in a recent study we investigated SPQ-B, a 22-item version of the SPQ, and found that Swedes rate themselves considerably lower than most other populations across 14 nations, worldwide [5]. Consequently, in a Swedish cultural context, individuals with ASD experience schizotypy symptoms to a high degree.

We found a substantial association between ASD and schizotypy among our participants, i.e., individuals with more autistic traits rated themselves as having more schizotypal traits as well. Considering how much the gestalt of schizotypy resembles ASD, this is not surprising. A nosological link between ASD and schizoid/schizotypal personality traits has been observed and debated for almost a century. Clinical descriptions of schizoid psychopathy in childhood [51], schizotypal personality disorders in children [39] and ‘schizoid’ personality of childhood [59] are all relevant to this. One reason for introducing the concept of schizotypy was to identify persons with elevated risk of psychosis. Accordingly, assessment instruments for schizotypy, including the SPQ, usually convey the psychopathological structure of schizophrenia, i.e., positive, negative, and disorganised symptoms. The negative symptoms of schizophrenia (and “negative schizotypy”) are generally viewed as closely related to schizoid personality and (at least superficially) very similar to core symptoms of ASD. The positive symptoms are usually believed to discriminate better between schizophrenia and ASD, which was supported by Spek & Wouters [50]. However, Barneveld et al. [3] argued that their ASD subjects had elevated measures on all three higher order constructs of the SPQ, to levels similar to patients with schizophrenia, and that all three were related to measures of autism. Based on a considerably larger sample of ASD subjects, our correlations of Positive and Negative, as well as Disorganised schizotypy with RAADS-14 corroborate the findings of Barneveld et al. However, when we take a closer look at the relationship between the SPQ-BR Positive schizotypy and Disorganized constructs, only two of their subscales (Suspiciousness and Eccentric behavior) showed a highly significant relationship with autistic traits, while no other subscales within these constructs did. This suggests that the overlap between ASD and schizotypy is mainly driven by Interpersonal schizotypy (i.e., the Negative schizotypy and the Social Anxiety constructs), supporting Spek & Wouters’ findings [50] (Table 2).

The diagnostic boundaries between autism and schizophrenia spectrum disorders are still unclear and different diagnostic traditions may come to different conclusions. High levels of schizotypy may balance the diagnosis towards a schizophrenia spectrum disorder and reversely, the prodromal schizophrenia state, characterized by a broad range of cognitive deficits that predate the onset of clinical symptoms, may suggest an autism. The considerable overlap between schizotypy and ASD needs to be considered. Prominent schizotypal traits in people with ASD may constitute an endophenotype coinciding with a particularly poor quality of life.

However, quality of life can improve through various forms of interventions [22, 41], thus adults with ASD ought not be disqualified because of high levels of schizotypy.

### Limitations

There are some limitations to our study. First, almost all data was collected through self-report. This may lead to unreliable responses due to e.g., misinterpretations. Although this limitation is well known, self-reports are regularly used in psychiatric research and in clinical practice. Hesselmark conducted a study investigating the reliability and validity of self-reported questionnaires in adults with ASD, and supported the use of these [23]. In fact, among individuals with ASD, self-reports could be preferred; it is presumably less stressful to sit peacefully by oneself and respond, without time limits and distractions, than being interviewed.

A second limitation was our skewed gender proportion with a majority of women. Although ASD is more common in men than in women, women tend to seek psychiatric treatments more often than men, which may explain the female preponderance in our study. Gender did not show any association with schizotypy scores and the gender distribution was very similar in the SPQ high and low group; thus, the gender bias should not have affected the results of our study.

Third, other researchers have suggested general health and social relationships to affect quality of life beyond depression [54]. Unfortunately, we did not use any measures to quantify general health or quality of social relationships in our study, which is another limitation. However, our participants were rather young, which makes them less likely to be affected by physical illness. Moreover, several items in the SPQ-BR concern social relationships; hence, this was intertwined in our assessment.

Fourth, we did not examine executive functioning. In a recent study, sustained attention differed between individuals with schizotypal personality disorder and individuals with ASD [1]. Preferably, clinical, and behavioural phenotypes should be investigated in the endeavour to delineate the underlying mechanisms in clinical populations with substantial phenotypical overlaps.

Fifth, we used a life quality questionnaire, the BBQ, which has not been validated in an autism sample. Ideally, we should have chosen the well-established World Health Organisation measure (WHOQoL-BREF) combined with autism-specific items (ASQoL) adapted for people with autism [38], however, the ASQoL was not available when we designed our study. We were concerned about the risk of dropouts if we set the threshold for inclusion too ambitiously. BBQ covers six domains in life and consists of 12 items whereas a measure such as the WHOQoL-BREF combined with ASQoL adds up to almost three times as many items. Furthermore, the BBQ resembles the more elaborate 32-item Quality of Life Inventory (QOLI, [17]). Both BBQ and QOLI measure life satisfaction rather than lack of symptoms and impairment. They ask about domains in life, which is followed by inquiring whether this domain is important to the person or not. In sum, BBQ and OQLI both ask if the person is satisfied with the parts of life that matter most, not what others may think is important. We believe this design enables inclusion of idiosyncrasies that are experienced by many autistic individuals. In a previous CBT study among adults with ASD [22], the participants did not express any difficulties using the QOLI, but the instrument was nevertheless regarded as too extensive to be included in the present internet-based study.

Sixth, the evidence for the validity of a schizotypy measure in autism populations could be questioned. However, in our post-hoc analysis we tried to separate schizotypy from autistic traits by excluding comorbid, mainly negative schizotypy traits. The impact of schizotypy still influenced symptoms of depression, anxiety, and quality of life. For the purpose of studying the validity of schizotypy constructs in ASD in future studies, the use of the full item versions of RAADS-R and SPQ would be preferable, rather than the abbreviated ones that we applied in the present study.

Finally, the ASD diagnosis was not confirmed in 14 out of the first 84 consecutive participants which could be considered as a major limitation. The reason for the lack of diagnostic confirmation was cumbersome administrative work and obstacles when ordering the diagnostic work-ups from clinics across the country. Although several reminders, not all clinics responded to our requests. When no signs suggested participants to falsely declare to have been diagnosed with ASD we decided not to check the correctness of the ASD diagnosis in the remaining 30 participants.

### Clinical implications

Our findings underscore the importance of identifying schizotypy in individuals with ASD. Schizotypy, anxiety and depressive symptoms, possibly inherent to ASD, contribute to the low quality of life often reported by people with ASD. By taking a broad approach towards the difficulties often experienced by people with ASD, instead of focusing on a limited area, we have reasons to believe that quality of life can be improved. This was done in an ambitious, group-based cognitive behavioural psychotherapy project (ALMA), resulting in improvements in quality of life among adult participants with ASD [4, 22].

## Conclusion

This study suggests that there is a considerable overlap between schizotypy and autism that needs to be considered. Prominent schizotypal traits in people with ASD may constitute an endophenotype coinciding with a particularly poor quality of life.

## Data Availability

Data and materials are available upon request.

## Abbreviations

ASD: Autism Spectrum Disorder
BBQ: The Brunnsviken Brief Quality of Life Scale
CBT: Cognitive Behavioural Therapy
EQ-5D-5L: European quality of life index version 5D (the 5-level version)
HADS: The Hospital Anxiety and Depression scale
RAADS-14 Screen: Ritvo Autism and Asperger Diagnostic Scale-Revised Screen 14 items
SPQ-BR: The Schizotypal Personality Questionnaire – Brief Revised

## References

[1] Abu-Akel A, Philip R, Lawrie SM, Johnstone EC, Stanfield AC (2020). Categorical and dimensional approaches to examining the joint effect of autism and schizotypal personality disorder on sustained attention. Front Psychiatry.

[2] APA (2013). Diagnostic and statistical manual of mental disorders.

[3] Barneveld PS, Pieterse J, de Sonneville L, van Rijn S, Lahuis B, van Engeland H, Swaab H (2011). Overlap of autistic and schizotypal traits in adolescents with autism Spectrum disorders. Schizophr Res.

[4] Bejerot S, Björnstjerna E (2019). ALMA – KBT för vuxna med autismspektrumsyndrom, manual och arbetsbok [ALMA-CBT for adults with autism spectrum disorder, manual and workbook].

[5] Bejerot S, Wallén J, Manouilenko I, Hesselmark E, Elwin M (2020). Schizotypal traits in Swedish speaking psychiatric patients and non-psychiatric controls. Nordic J Psychiatry.

[6] Callaway DA, Cohen AS, Matthews RA, Dinzeo T (2014). Schizotypal personality questionnaire—brief revised: psychometric replication and extension. Personal Disord Theory Res Treat.

[7] Chisholm K, Lin A, Abu-Akel A, Wood SJ (2015). The association between autism and schizophrenia spectrum disorders: a review of eight alternate models of co-occurrence. Neurosci Biobehav Rev.

[8] Cicero DC, Jonas KG, Li K, Perlman G, Kotov R (2019). Common taxonomy of traits and symptoms: linking schizophrenia symptoms, Schizotypy, and Normal personality. Schizophr Bull.

[9] Cohen AS, Davis TE (2009). Quality of life across the schizotypy spectrum: findings from a large nonclinical adult sample. Compr Psychiatry.

[10] Cohen AS, Matthews RA, Najolia GM, Brown LA (2010). Toward a more psychometrically sound brief measure of schizotypal traits: introducing the SPQ-brief revised. J Personal Disord.

[11] Crespi B, Dinsdale N, Read S, Hurd P (2019). Spirituality, dimensional autism, and schizotypal traits: the search for meaning. PLoS ONE.

[12] Davidson CA, Hoffman L, Spaulding WD (2016). Schizotypal personality questionnaire--brief revised (updated): an update of norms, factor structure, and item content in a large non-clinical young adult sample. Psychiatry Res.

[13] Dinsdale NL, Hurd PL, Wakabayashi A, Elliot M, Crespi BJ (2013). How are autism and schizotypy related? Evidence from a non-clinical population. PLoS ONE.

[14] Eriksson JM, Andersen LM, Bejerot S. RAADS-14 screen: validity of a screening tool for autism spectrum disorder in an adult psychiatric population. Mol Autism. 2013;4(49). 10.1186/2040-2392-4-49. 10.1186/2040-2392-4-49 PMC3907126 24321513

[15] Evans B (2013). How autism became autism. Hist Hum Sci.

[16] Fonseca-Pedrero E, Cohen A, Ortuño-Sierra J, de Álbeniz AP, Muñiz J (2017). Dimensional structure and measurement invariance of the schizotypal personality questionnaire - brief revised (SPQ-BR) scores across American and Spanish samples. J Personal Disord.

[17] Frisch MB, Cornell J, Villanueva M, Retzlaff PJ (1992). Clinical validation of the quality of life inventory. A measure of life satisfaction for use in treatment planning and outcome assessment. Psychol Assess.

[18] González-Blanch C, Hernández-de-Hita F, Muñoz-Navarro R, Ruíz-Rodríguez P, Medrano LA, Cano-Vindel A. The association between different domains of quality of life and symptoms in primary care patients with emotional disorders. Sci Rep. 2018;8. 10.1038/s41598-018-28995-6. 10.1038/s41598-018-28995-6 PMC6060102 30046118

[19] Grant P, Green MJ, Mason OJ (2018). Models of Schizotypy: the importance of conceptual clarity. Schizophr Bull.

[20] Hayes AF (2009). Beyond Baron and Kenny: statistical mediation analysis in the new millennium. Commun Monogr.

[21] Herdman M, Gudex C, Lloyd A, Janssen M, Kind P, Parkin D, Bonsel G, Badia X (2011). Development and preliminary testing of the new five-level version of EQ-5D (EQ-5D-5L). Qual Life Res Int J Qual Life Asp Treat Care Rehab.

[22] Hesselmark E, Plenty S, Bejerot S (2013). Group cognitive behavioural therapy and group recreational activity for adults with autism spectrum disorders: a preliminary randomized controlled trial. Autism.

[23] Hesselmark E, Eriksson JM, Westerlund J, Bejerot S (2015). Autism spectrum disorders and self-reports: testing validity and reliability using the NEO-PI-R. J Autism Dev Disord.

[24] Hsu N-W, Tsao H-M, Chen H-C, Chou P (2014). Anxiety and depression mediate the health-related quality of life differently in patients with cardiovascular disease and stroke–preliminary report of the Yilan study: a population-based community health survey. PLoS ONE.

[25] Janssen B, Szende A, Szende A (2013). Population norms for the EQ-5D. Self-reported population health: an international perspective based on EQ-5D.

[26] Joshi, G., Wozniak, J., Petty C., Martelon, M. K, Fried, R., Bolfek, A., Kotte, A.,Stevens, J., Furtak, S., Bourgeois, M., Cauruso, J., & Caron A. (2013). Psychiatric comorbidity and functioning in a clinically referred population of adults with autism spectrum disorders: a comparative study. J Autism Dev Disord, 43(6), 1314–1325, 10.1007/s10803-012-1679-5. 10.1007/s10803-012-1679-5 23076506

[27] Katschnig H (2006). Quality of life in mental disorders: challenges for research and clinical practice. World Psychiatry: Official Journal of the World Psychiatric Association (WPA).

[28] Kessler RC, Chiu WT, Demler O, Merikangas KR, Walters EE (2005). Prevalence, severity, and comorbidity of 12-month DSM-IV disorders in the National Comorbidity Survey Replication. Arch Gen Psychiatry.

[29] Kwapil TR, Gross GM, Burgin CJ, Raulin ML, Silvia PJ, Barrantes-Vidal N (2018). Validity of the multidimensional Schizotypy scale: associations with schizotypal traits and normal personality. Personal Disord.

[30] Lai MC, Kassee C, Besney R, Bonato S, Hull L, Mandy W, Szatmari P, Ameis SH (2019). Prevalence of co-occurring mental health diagnoses in the autism population: a systematic review and meta-analysis. Lancet Psychiatry.

[31] Lindner P, Frykheden O, Forsström D, Andersson E, Ljótsson B, Hedman E, Andersson G, Carlbring P (2016). The Brunnsviken brief quality of life scale (BBQ): development and psychometric evaluation. Cogn Behav Ther.

[32] Lisspers J, Nygren A, Söderman E (1997). Hospital anxiety and depression scale (HAD): some psychometric data for a Swedish sample. Acta Psychiatr Scand.

[33] Lord C, Elsabbagh M, Baird G, Veenstra-Vanderweele J (2018). Autism spectrum disorder. Lancet (London, England).

[34] Lugnegård T, Hallerbäck MU, Gillberg C (2012). Personality disorders and autism spectrum disorders: what are the connections?. Compr Psychiatry.

[35] Martino G, Catalano A, Bellone F, Russo GT, Vicario CM, Lasco A, Quattropani MC, Morabito N (2019). As time Goes by: anxiety negatively affects the perceived quality of life in patients with type 2 diabetes of long duration. Front Psychol.

[36] Mason OJ (2014). The duality of Schizotypy: is it both dimensional and categorical?. Front Psychiatry.

[37] Mason D, McConachie H, Garland D, Petrou A, Rodgers J, Parr JR (2018). Predictors of quality of life for autistic adults. Autism Res.

[38] McConachie H, Mason D, Parr JR, Garland D, Wilson C, Rodgers J (2018). Enhancing the validity of a quality of life measure for autistic people. J Autism Dev Disord.

[39] Nagy J, Szatmari P (1986). A chart review of schizotypal personality disorders in children. J Autism Dev Disord.

[40] Oliver LD, Moxon-Emre I, Lai MC, Grennan L, Voineskos AN, Ameis SH (2020). Social cognitive performance in schizophrenia Spectrum disorders compared with autism spectrum disorder: a systematic review, Meta-analysis, and Meta-regression. JAMA Psychiatry.

[41] Pallathra AA, Cordero L, Wong K, Brodkin ES (2019). Psychosocial interventions targeting social functioning in adults on the autism Spectrum: a literature review. Curr Psychiatry Rep.

[42] Pisula E, Danielewicz D, Kawa R, Pisula W (2015). Autism spectrum quotient, coping with stress and quality of life in a non-clinical sample - an exploratory report. Health Qual Life Outcomes.

[43] Raine A (1991). The SPQ: a scale for the assessment of schizotypal personality based on DSM-III-R criteria. Schizophr Bull.

[44] Rapaport MH, Clary C, Fayyad R, Endicott J (2005). Quality-of-life impairment in depressive and anxiety disorders. Am J Psychiatry.

[45] Ritvo RA, Ritvo ER, Guthrie D, Ritvo MJ, Hufnagel DH, McMahon W, Tonge B, Mataix-Cols D, Jassi A, Attwood T, Eloff J (2011). The Ritvo autism Asperger diagnostic scale-revised (RAADS-R): A scale to assist the diagnosis of autism Spectrum disorder in adults: an international validation study. J Autism Dev Disord.

[46] Ruzich E, Allison C, Smith P, Watson P, Auyeung B, Ring H, et al. Measuring autistic traits in the general population: a systematic review of the autism-Spectrum quotient (AQ) in a nonclinical population sample of 6,900 typical adult males and females. Mol Autism. 2015;6(2). 10.1186/2040-2392-6-2. 10.1186/2040-2392-6-2 PMC4396128 25874074

[47] Rydén E, Bejerot S (2008). Autism spectrum disorder in an adult psychiatric population. A naturalistic cross sectional controlled study. Clinical Neuropsychiatry: Journal of Treatment Evaluation.

[48] Schonfeld WH, Verboncoeur CJ, Fifer SK, Lipschutz RC, Lubeck DP, Buesching DP (1997). The functioning and well-being of patients with unrecognized anxiety disorders and major depressive disorder. J Affect Disord.

[49] Son YJ, Choi KS, Park YR, Bae JS, Lee JB (2009). Depression, symptoms and the quality of life in patients on hemodialysis for end-stage renal disease. Am J Nephrol.

[50] Spek A, Wouters S (2010). Autism and schizophrenia in high functioning adults: behavioral differences and overlap. Res Autism Spectr Disord.

[51] Sucharewa GE (1926). Die schizoiden Psychopathien im Kindesalter. Monatsschr Psychiatr Neurol.

[52] Ten Hoopen LW, de Nijs P, Duvekot J, Greaves-Lord K, Hillegers M, Brouwer W, Hakkaart-van Roijen L (2020). Children with an autism Spectrum disorder and their caregivers: capturing health-related and care-related quality of life. J Autism Dev Disord.

[53] van Heijst BF, Geurts HM (2015). Quality of life in autism across the lifespan: a meta-analysis. Autism Int J Res Pract.

[54] Villas-Boas S, Oliveira AL, Ramos N, Montero I (2019). Predictors of quality of life in different age groups across adulthood: research. J Intergenerational Relatsh.

[55] Watson HJ, Swan A, Nathan PR (2011). Psychiatric diagnosis and quality of life: the additional burden of psychiatric comorbidity. Compr Psychiatry.

[56] Westen D, Shedler J (1999). Revising and assessing axis II, part II: toward an empirically based and clinically useful classification of personality disorders. Am J Psychiatry.

[57] WHO, World Health Organization (1993). The ICD-10 classification of mental and behavioural disorders: diagnostic criteria for research.

[58] WHO, World Health Organization (2020) ICD-11 - Mortality and Morbidity Statistics [Internet]. [cited 2021 Jan 7]; Available from https://icd.who.int/browse11/l-m/en#/http://id.who.int/icd/entity/437815624;https://icd.who.int/browse11/l-m/en#/ and http://id.who.int/icd/entity/18178000.

[59] Wolff S (1991). 'Schizoid' personality in childhood and adult life. I: the vagaries of diagnostic labelling. Br J Psychiatry J Ment Sci.

[60] Zigmond AS, Snaith RP (1983). The hospital anxiety and depression scale. Acta Psychiatr Scand.

